# Anti-PLA2R1 Antibodies as Prognostic Biomarker in Membranous Nephropathy

**DOI:** 10.1016/j.ekir.2021.04.002

**Published:** 2021-04-22

**Authors:** Anne-Els van de Logt, Joana Justino, Coralien H. Vink, Jan van den Brand, Hanna Debiec, Gérard Lambeau, Jack F. Wetzels

**Affiliations:** 1 Radboud University Medical Center, Radboud Institute for Health Sciences, Department of Nephrology, Nijmegen, The Netherlands; 2 Université Côte d’Azur, Centre National de la Recherche Scientifique, Institut de Pharmacologie Moléculaire et Cellulaire, UMR7275 Valbonne Sophia Antipolis, France; 3 Institut National de la Santé et de la Recherche Médicale (INSERM), UMR-S1155, Paris, France

**Keywords:** anti-PLA2R1 antibodies, glomerulonephritis, membranous nephropathy

## Abstract

**Introduction:**

Personalized treatment for patients with membranous nephropathy requires accurate prediction of the disease course at an early stage. In this study, we evaluated the value of baseline anti–phospholipase A2 receptor (PLA2R1) antibody titer as a prognostic biomarker in patients with PLA2R1-associated membranous nephropathy.

**Methods:**

In this cohort study, we included 168 patients (118 men, 50 women) referred to our nephrology center between February 1995 and November 2016. Mean age was 52 ± 13 years. There were 156 patients with new-onset disease and 12 patients with a relapse (*n* = 10) or recent use of immunosuppressive therapy (*n* = 2). We measured anti-PLA2R1 titer at baseline and analyzed progression to severe disease (30% increase of serum creatinine or start of immunosuppressive therapy) as a primary study endpoint over 60 months.

**Results:**

There was a clear association between anti-PLA2R1 antibody titer and severity of the nephrotic syndrome. In univariate analysis, anti-PLA2R1 antibody titer was also associated with disease progression. However, in Cox proportional hazard models that included proteinuria and serum creatinine, anti-PLA2R1 antibody titer was no longer associated with clinical outcome. Results were similar when limiting the analysis to the patients with new-onset disease.

**Conclusion:**

Our study questions the relevance of single measurement of anti-PLA2R1 antibodies at baseline as a prognostic biomarker in membranous nephropathy. Future studies are needed to determine the possible role of sequential measurements of anti-PLA2R1 antibodies as a prognostic biomarker of disease progression.

Primary membranous nephropathy is the most common cause of nephrotic syndrome in nondiabetic Caucasian adults.^1^ Primary membranous nephropathy (PMN) is considered a renal-limited autoimmune disease with circulating autoantibodies against the phospholipase A2 receptor (aPLA2R1ab) present in 70% to 80% of patients.^2^^,^^3^ After 5 to 10 years of observation, the natural course of the disease is variable, with 40% to 50% of untreated patients progressing to end-stage renal disease, whereas the remaining patients enter into spontaneous remission, sometimes after years of stable but persistent proteinuria.^4^^,^^5^

Personalized treatment for PMN patients requires accurate prediction of the disease course at an early stage, before the start of immunosuppressive treatment. The best known biomarkers are the magnitude and duration of proteinuria, and deterioration of kidney function, reflected by increased serum creatinine or changes in creatinine clearance.^6^ These biomarkers were combined in the validated “Toronto risk score.”^6^ We recently validated urinary excretion of low molecular weight proteins including urine alpha-1-microglobulin as prognostic biomarker.^5^ Unfortunately, accuracy of current prognostic biomarkers is at best 80%.^5^

Recent studies suggested that measurement of aPLA2R1ab titer may have added value. Indeed, titer of circulating aPLA2R1ab correlated with clinical disease activity.^7^ Additional studies suggested their value as a prognostic biomarker, because low titers of aPLA2R1ab were associated with a higher likelihood of remission.^8^, ^9^, ^10^, ^11^, ^12^ The discovery of aPLA2R1ab associated with different epitope profiles in patients (due to epitope spreading and defining "spreaders" and "nonspreaders") also received considerable interest. Two studies concluded that epitope spreading at baseline was associated with clinical outcome.^13^^,^^14^ A more recent study raised concerns about the use of epitope profile as an independent biomarker with added value over aPLA2R1ab titers.^15^

Still, the preceding studies have limitations. The studied cohorts were rather small. Moreover, remission and renal progression rates were sometimes evaluated in a mixed cohort of treated and untreated patients. When using biomarkers, it is important that the study protocols define the specific use of a biomarker. A recent Food and Drug Administration guidance document distinguishes between prognostic biomarkers (i.e., biomarkers that allow the selection of patients at risk for severe disease progression), and predictive biomarkers that are used to determine the effectiveness of treatment.^16^ Thus, it remains unclear whether the measurement of aPLA2R1ab titers or epitope spreading is of prognostic value (predicting the clinical evolution during the natural course of disease in untreated patients) or of predictive value (predicting response to immunosuppressive therapy).

For the first time, this study evaluated the prognostic value of aPLA2R1ab titers at baseline next to the traditional biomarkers (proteinuria, serum creatinine) in a large cohort of patients with PLA2R1-associated MN.

## Methods

### Patients

Since 1995, patients with PMN referred to our hospital were evaluated in a standardized way.^17^ Written informed consent was obtained and the study was performed in accordance to the Declaration of Helsinki. Serum samples were collected and stored at −80°C. For this study, we included patients with PMN and nephrotic range proteinuria (urine protein-creatinine ratio [UPCR] ≥ 3 g/10 mmol in 24-hour urine), with preserved renal function (serum creatinine level below 135 μmol/l). Secondary causes of MN were excluded according to our standard policy.^17^ To evaluate the role of aPLA2R1ab titers and epitope spreading as a biomarker of prognosis, we selected all patients who were identified as positive for aPLA2R1ab, based on our in-house IgG4 anti-PLA2R1 enzyme-linked immunosorbent assay (ELISA).^13^

All patients were treated to decrease blood pressure (target value 130/80 mm Hg), primarily by using angiotensin-converting-enzyme inhibitors/angiotensin receptor blockers (ACEi/ARBs). Anticoagulant drugs were advised when serum albumin levels (immunonephelometric assay) dropped below 20 g/l. Patients were treated according to our restrictive treatment strategy.^18^ Thus, patients were followed at the outpatient clinic, while waiting for spontaneous remission. Immunosuppressive therapy (oral cyclophosphamide 1.5 mg/kg daily in combination with prednisolone during 6–12 months in most patients) was only prescribed to patients with an increase in serum creatinine level ≥30% or patients with (complications of) a severe disabling nephrotic syndrome.

### Definitions and Calculations

To correct for inappropriate 24-hour urine collection, proteinuria was expressed as UPCRs (UPCR in grams per 10 mmol of creatinine). In the patient population, the average creatinine output is 12 mmol/d. Therefore, we choose 3 g/10 mmol creatinine, which equals 3.5 g/d, which is used in the definition of remission according to the Kidney Disease: Improving Global Outcomes guideline.^19^ Nephrotic syndrome was defined as UPCR >3 g/10 mmol/l and serum albumin <30 g/l. Complete remission was defined as UPCR ≤0.2 g/10 mmol creatinine with stable kidney function, and partial remission was defined as UPCR <3 g/10 mmol creatinine with a reduction of at least 50% from baseline and stable kidney function (≤ 30% increase of serum creatinine level from baseline). Achieving spontaneous remission includes both partial and complete remission. Time from biopsy was calculated as the time between kidney biopsy and the first standardized evaluation in our center.^18^ Follow-up duration was calculated from this first standardized evaluation onward.

### Clinical Outcome

Patients were followed at regular intervals according to local practice. Progression to severe disease was considered as the primary study endpoint. Patients with progressive disease (referred to as progressors) were defined as those requiring immunosuppressive therapy because of an increase of serum creatinine level >30% from baseline or severe persistent nephrotic syndrome. Spontaneous remission was defined as remission occurring while on conservative therapy only. For this analysis, patients were censored at the time they reached the endpoint. The endpoint was defined only after repeated confirmatory measurements. We investigated a range of prediction horizons up to 60 months. We limited to 60 months, because we have shown that >95 % of patients have reached an endpoint within 5 years after disease onset.^18^

### Anti-PLA2R1 Antibody Assays

Stored serum samples were retrieved from the Radboud biobank^20^ and assessed for aPLA2R1ab titer. Patients were considered aPLA2R1ab positive if aPLA2R1abs were positive in our in-house IgG4 anti-PLA2R1 ELISA,^13^ which was more sensitive than the standardized commercial ELISA. Titers for anti-PLA2R1ab were quantified with the standardized commercial ELISA (Euroimmun, Lübeck, Germany).^21^ Epitope spreading defined by positivity for autoantibodies targeting specific epitopes in the CysR, CTLD1, or CTLD7 domains were measured by HA-capture ELISA on 96-well plates coated with CysR-HA, CTLD1-HA, CTLD7-HA single domains or mock medium from HEK293-transfected cells as previously described.^13^

### Anti-PLA2R1ab Level and Prognosis in the Conservative Treatment Arm of the GEMRITUX Trial

We confirmed our findings in 26 patients with PLA2R1-associated MN, included in the control arm of the GEMRITUX clinical trial. Details of patients’ inclusion and characteristics have been published.^22^ Of the original study, 26 of 38 (68%) patients could be included, because not all patients gave informed consent for data sharing.

### Statistical Analyses

Data are presented as frequency (percentage), mean (±SD), or median (interquartile ranges [IQR]) when appropriate. T-test, Mann-Whitney *U* test, and χ^2^ test were used for comparisons between and within groups.

There were very few missing values in the variables of interest, therefore we performed a complete case analysis without imputation. First, we fitted a univariate generalized additive model based on the Cox proportional hazards model and plotted the predicted values to visualize the dose-response relationship between predictors and the respective clinical outcomes. Based on these plots, we took the natural log for aPLA2R1ab titers. In the Cox proportional hazard model, we included serum creatinine and UPCR in a reference model. Next, we added baseline aPLA2R1ab and/or epitope spreading in the following models. Subsequently, we analyzed different cutoff values for the independent prognostic factors and calculated sensitivity, specificity, and accuracy for predicting the outcome of progression. If a patient achieved spontaneous remission, patients were censored from that moment on for the outcome of progression. Accuracy was calculated as the number of correctly classified patients (true positives and true negatives, divided by the total number of patients).

We determined model fit using the pseudo *R*^2^. The discriminative ability of the model was determined by taking the area under the receiver operating characteristic curve at follow-up times of 6, 12, 18, 24, 36, and 60 months. The calibration between predicted and observed risk was assessed at these time points as well as using calibration plots.

Analyses were performed with SPSS version 25 (IBM Corp., Armonk, NY) and R with the RStudio shell (R Version 3.5.1, RStudio version 1.1463), and packages foreign_0.8–70, dplyr_0.8.0.1, ggplot2_3.1.1, gridExrta_2.3, tableone_0.10.0, mgcv_1.8–24, crrstep_2015–2.1, survival_2.42–3, riskRegression_2019.01.29 and their dependencies. Differences were considered significant with *P* < 0.05.

## Results

**Figure 1:**
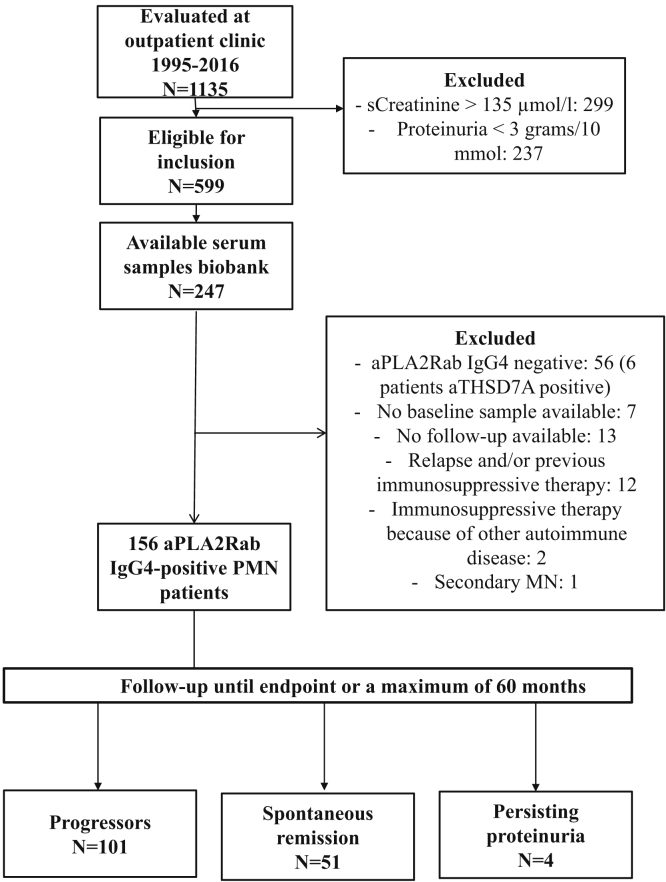
Flowchart of patients' inclusion. MN, membranous nephropathy.

**Figure 2:**
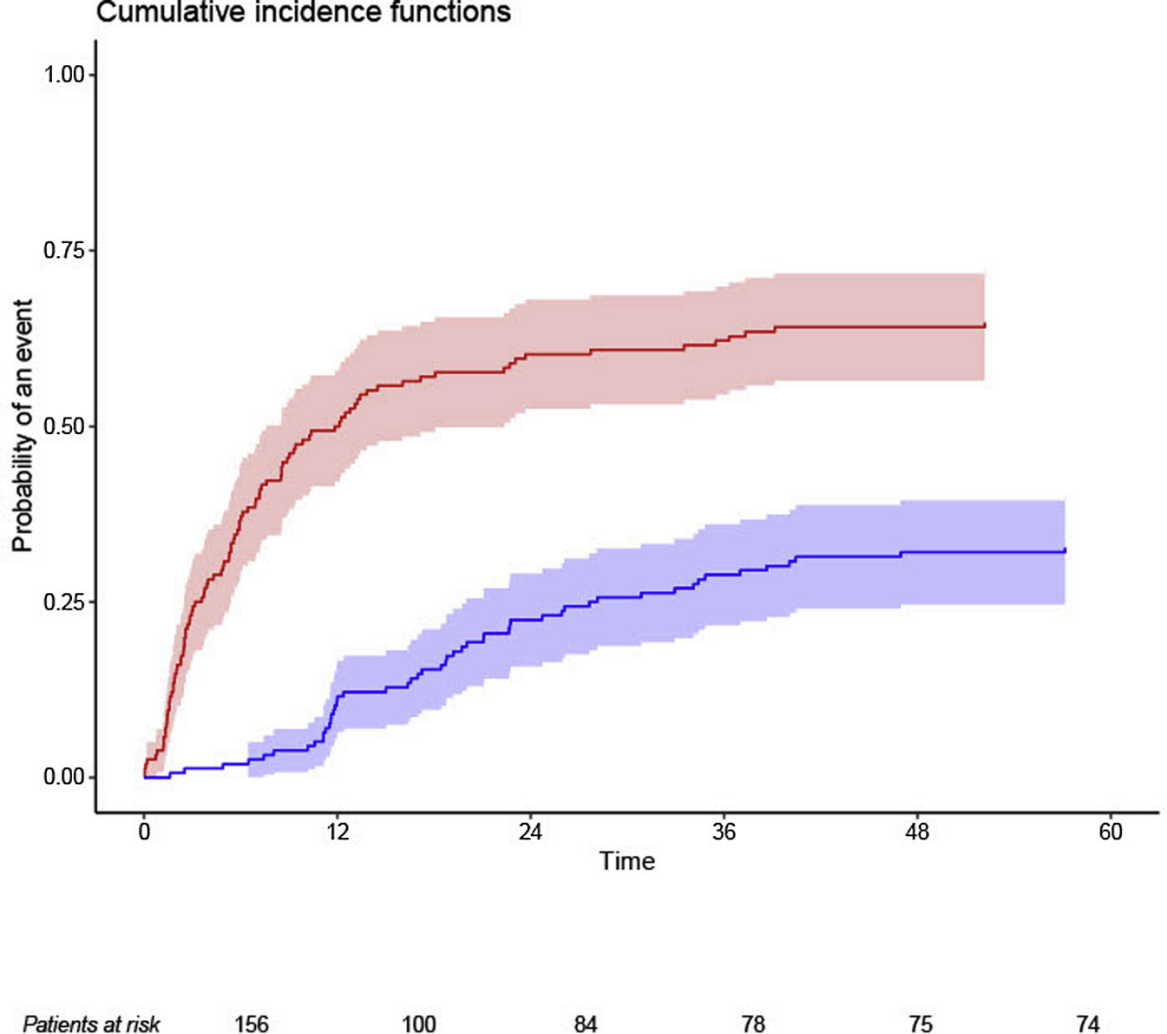
The cumulative incidence of progression (red line) and spontaneous remission (blue line). The number of patients at risk of progression is presented on the X-axis.

In the period from February 1995 to November 2016, 1135 patients with PMN were seen at our outpatient clinic. Of these, 599 patients fulfilled the inclusion criteria and of 247 patients samples were available in our biobank (Figure 1). From these 247 patients, 168 were aPLA2R1ab positive and finally included in this study. Of these, 64 patients were included in a previously described cohort.^18^ In 156 patients, MN was newly diagnosed. Twelve patients were evaluated with a documented relapse (*n* = 10), or recent use of immunosuppressive therapy (*n* = 2). In our initial analysis we included all 168 patients. We observed lower aPLA2R1ab levels in these 12 patients (median aPLA2R1ab titer 45 RU/ml [IQR 12–86 RU/ml]). Therefore, a second analysis including only patients with incident disease was done. Results of all 168 patients are given in Supplementary Appendix Part A Tables 1-4. The baseline characteristics of the 156 incident patients are described in Table 1. The interval between kidney biopsy and the standardized evaluation at our center was 2.3 months. At the time of evaluation, 85% of patients were on ACEi/ARBs. All patients were followed until the endpoint, or a maximum of 60 months. During the 60 months of follow-up, 152 patients reached the study endpoint, that is, disease progression (*n* = 101, 65%) or spontaneous remission (*n* = 51, 33%). Four patients (2%) had persisting proteinuria. Progression occurred within a median of 5 months [IQR 2–11] after initial evaluation. Spontaneous remission occurred within a median of 18 months [IQR 11–28] after initial evaluation. Complete spontaneous remission was achieved in 21 patients (40% of all remissions) within a median of 51 months [IQR 35–77] after initial evaluation. The cumulative incidence of progression versus spontaneous remission is depicted in Figure 2. We compared patients who progressed with those developing spontaneous remission or with persisting proteinuria (Table 1). Age, gender, and time from biopsy were not different between groups. Progressors had higher serum creatinine, a more severe nephrotic syndrome, higher levels of urinary low molecular weight proteins, higher titers of aPLA2R1ab, were more often “spreaders,” and used ACEi/ARB therapy less often.

**Table 1:** Risk characteristics of patients with new-onset primary membranous nephropathy according to outcome during the 60-month observation period

Clinical characteristics	Overall (*n* = 156)	Progressors *n* = 101	Remission *n* = 51	Persistent NS *n* = 4^a^	*P* value
Age (y)	52 ± 13	53 ± 13	51 ± 13	45 [40–61]	0.470
Gender (M/F, % M)	112/44 (72)	71/30 (70)	37/14 (73)	4/0 (100)	0.428
Time from biopsy (mo) b	2.3 [1.2–6.6]	2.2 [1.1–5.5]	2.8 [1.4–7.8]	9.9 [0.9–31]	0.198
Serum creatinine (μmol/l)	92 ± 18	96 ± 19	86 ± 15	94 [69–116]	**0.009**
Serum albumin (g/l)	21 ± 5	19 ± 5	23 ± 5	24 [21–28]	**0.000**
Serum cholesterol (mmol/l)	6.6 [5.2–8.7]	7.3 [5.5–9.3]	5.8 [5.2–7.1]	6.0 [5.0–13.0]	**0.005**
Serum IgG (g/l)	4.4 [3.1–5.4]	3.8 [2.9–5.1]	5.0 [4.0–6.1]	4.0 [4.0–8.0]	**0.005**
Proteinuria (g/10 mmol)	7.2 [5.7–10.9]	9.4 [6.4–11.6]	6.0 [4.7–7.9]	5.5 [4.0–7.0]	**0.000**
aPLA2R1ab titer Euroimmun (RU/ml)	110 [47–244]	152 [70–312]	50 [33–128]	217 [39–829]	**0.000**
Spreaders, *n* (%)	112 (72)	81 (79)	29 (57)	2 (50)	**0.014**
Urinary β2m (ng/min)	981 [284–3808]	2257 [481–8626]	315 [168–692]	348 [126–8743]	**0.000**
Urinary α1m (μg/min)	46 [27–83]	63 [41–105]	29 [20–43]	13 [8–57]	**0.000**
IgG excretion (mg/24 h)	306 [165–504]	394 [237–600]	185 [108–314]	90 [60–309]	**0.000**
MAP (mm Hg)	93 [85–102]	93 [87–102]	90 [83–101]	95 [91–105]	0.299
ACEi/ARB use (%)	105/124 (85)	62/80 (78)	42/43 (98)	4/4 (100)	**0.002**

α1m, alpha-1-microglobulin; ACEi/ARB, angiotensin-converting-enzyme inhibitor/angiotensin receptor blockers; MAP, mean arterial blood pressure; NS, nephrotic syndrome.

Values are given as mean ± SD, median [IQR], number (percentage).

a In patients with persisting proteinuria (*n* = 4), values are given as median [range].

b Time from biopsy = interval between biopsy and standardized measurement in our hospital.

### Associations of aPLA2R1ab Titer and Epitope Spreading With Clinical Outcome

Baseline characteristics according to aPLA2R1ab tertiles are presented in Table 2. Age, gender, and interval between biopsy and standardized measurement were not different between groups. Patients in the highest tertile of aPLA2R1ab had a more severe nephrotic syndrome and a higher level of urinary low molecular weight proteins. There was a clear association between aPLA2R1ab titers and the prevalence of epitope spreading. In this cohort, all patients with aPLA2R1ab titer >176 RU/ml were spreaders. Progression was observed in 23 (44%), 37 (71%), and 43 (83%) of patients in the first, second, and third tertiles, respectively. The median time to progression was 4 months [IQR: 2–12], 6 months [IQR 2–13], and 5 months [IQR 2–10], respectively. Baseline characteristics of patients according to epitope spreading (spreaders versus nonspreaders) are given in Supplementary Appendix Part B Table S1. Progression was less frequent in nonspreaders (48%) than spreaders (73%).

**Table 2:** Baseline characteristics of patients according to tertiles of aPLA2R1ab

*n* = 156	First tertile^a^ 8–59 RU/mL *n* = 52	Second tertile 63–178 RU/mL *n* = 52	Third tertile 182–1239 RU/mL *n* = 52	*P* value
Age (y)	50 ± 11	53 ± 15	54 ± 12	0.564
Gender (M/F, % M)	39/13 (75)	38/14 (73)	35/17 (67)	0.663
Time from biopsy (months)	3.1 [1.4–8.7]	2.0 [1.1–5.9]	2.3 [1.2–6.6]	0.292
Serum creatinine (μmol/l)	90 [81–105]	91 [82–105]	95 [78–111]	0.208
Serum albumin (g/l)	24 [18–28]	20 [17–24]	19 [15–22]	0.063
Serum cholesterol (mmol/l)	6.3 [5.0–8.2]	6.8 [5.4–8.3]	6.7 [5.4–9.7]	0.584
Serum IgG (g/l)	4.9 [3.8–5.8]	3.9 [3.1–5.0]	4.2 [3.0–5.1]	0.199
Proteinuria (g/10 mmol)	6.0 [4.5–8.7]	8.0 [6.2–11.0]	9.0 [6.0–11.2]	**0.003**
Urinary β2m (ng/min)	547 [242–2298]	832 [202–3569]	2112 [382–8385]	**0.037**
Urinary α1m (μg/min)	42 [25–68]	49 [24–95]	58 [37–98]	0.070
IgG excretion (mg/24 h)	265 [122–391]	306 [120–557]	383 [229–591]	**0.009**
MAP (mm Hg)	93 [85–100]	93 [86–104]	92 [85–104]	0.886
ACEi/ARB use, *n* (%)	39/41 (95)	29/41 (71)	37/42 (88)	0.063
Spreaders, *n* (%)	29 (56)	37 (71)	46 (89)	**0.001**

α1m, alpha-1-microglobulin; ACEi/ARB, angiotensin-converting-enzyme inhibitor/angiotensin receptor blockers; MAP, mean arterial blood pressure.

Values are given as mean ± SD, median [IQR].

a There were 3 patients with an Euroimmun aPLA2Rab titer < 14 Ru/ml with a positive results in our homemade IG4 aPLA2Rab assay.

### Cox Proportional Hazard Models

Hazard ratios and C-statistics are given for the risk prediction models using a time horizon of 36 months in Table 3. Full data for all evaluated time horizons are given in Supplementary Appendix Part B Table S2. Proteinuria and serum creatinine were clear prognostic biomarkers in a model using only these 2 biomarkers (model 1, area under the curve [AUC] 0.715). Anti-PLA2R1ab did not improve the model (model 2 AUC 0.708). Epitope spreading as an additional single variable slightly improved model 1 (model 3 AUC 0.723 Supplementary Appendix Part B Table S3). Calibration plots are given in the supplementary appendix (Supplementary Appendix Part B Figure S1).

**Table 3:** Cox regression analysis: models with known biomarkers for the prediction of progression

	AUC 36 mo	HR	95% CI
**Model 1**			
Screat	0.715	1.019	1.007–1.031
UPCR		1.185	1.116–1.258
**Model 2**			
Screat	0.708	1.018	1.006–1.030
UPCR		1.175	1.105–1.249
Log.aPLA2R1ab		1.373	0.897–2.102

AUC, area under the curve/C-statistic; CI, confidence interval; HR, hazard ratio; Log.aPLA2R1ab, natural log of anti-PLA2R antibodies; Screat, serum creatinine; UPCR= protein-creatinine ratio in 24-hours urine.

### Individualized Risk Prediction: Test Characteristics of Prognostic Markers

Accuracy of prediction models is limited. The use of a model is not easy in clinical practice. To discuss management strategies with the individual patient, we provide the test characteristics of aPLA2R1ab for the prediction of progression (Supplementary Appendix Part B Table S4a). This table provides information that can be used to discuss prognosis with the individual patient based on the results of the biomarker measurement in that patient.

### Subanalysis in Patients With Normal Serum Creatinine Levels

**Figure 3:**
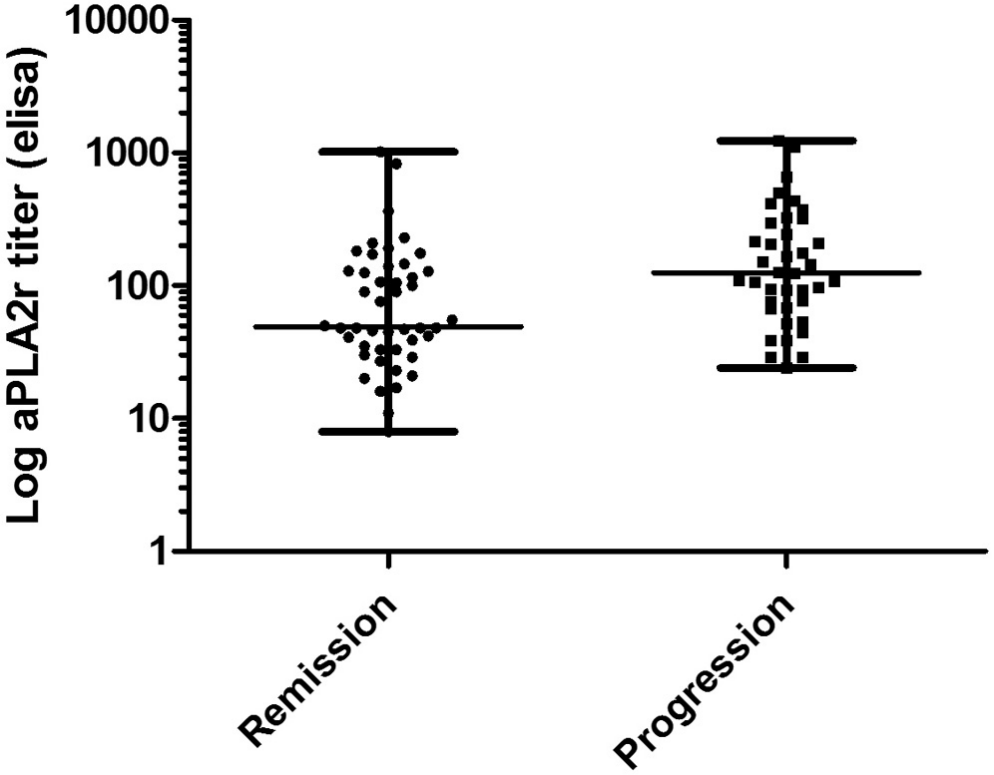
aPLA2R1ab levels at baseline of patients with normal serum creatinine with progression (*n* = 39) and or spontaneous remission (*n* = 46).

For this subanalysis, we excluded all female patients with a serum creatinine level ˃90 μmol/l and all male patients with a serum creatinine level ˃110 μmol/l. We also excluded patients who received immunosuppressive therapy within 6 months after standardized measurement. Of the original 156 patients, 85 patients remained for this analysis (Table 4). The number of progressors according to outcome based on total follow-up duration was 39 (46 %) with no differences between women and men. In univariate analysis aPLA2R1ab titers were numerically higher in patients with progressive disease, although not significant (*P* = 0.066). Also in this subgroup analysis, aPLA2R1ab levels did not improve a model incorporating serum creatinine and proteinuria. The limited value of aPLA2R1ab for individual patient care is shown in Figure 3, illustrating the wide overlap in aPLA2R1ab titers between progressors and nonprogressors. The test characteristics of aPLA2R1ab for the prediction of progression in this cohort is presented in Supplementary Appendix Part B Table S4b.

**Table 4:** Clinical characteristics of patients with normal serum creatinine at baseline

Clinical characteristics	Overall (*n* = 85)	Progressors *n* = 39	No progressors *n* = 46	*P* value
Age (y)	50 ± 12	51 ± 11	50 ± 13	0.674
Gender (M/F, % M)	65/20 (77)	30/9 (77)	35/11 (76)	1.000
Time from biopsy (mo)	2.1 [1.3–5.9]	2.1 [1.4–3.0]	2.1 [1.3–7.6]	0.892
Serum creatinine (μmol/l)	83 ± 13	85 ± 14	82 ± 12	0.463
Serum albumin (g/l)	22 ± 5	20 ± 5	23 ± 5	**0.038**
Serum cholesterol (mmol/l)	6.9 [5.3–8.9]	8.0 [6.2–9.5]	6.0 [5.2–7.5]	**0.004**
Serum IgG (g/l)	4.8 [3.4–5.6]	4.2 [3.0–5.3]	5.0 [4.0–6.2]	0.101
Proteinuria (g/10 mmol)	6.4 [4.9–9.3]	7.2 [5.6–11.0]	5.9 [4.6–7.7]	0.332
aPLA2R1ab titer (RU/ml)	95 [43–180]	125 [69–300]	49 [33–131]	0.066
Spreaders, *n* (%)	52/33 (61)	27 (69)	25 (54)	0.186
Urinary β2m (ng/min)	390 [178–1235]	550 [274–1686]	277 [154–553]	**0.035**
Urinary α1m (μg/min)	32 [22–50]	43 [29–67]	28 [14–42]	**0.023**
IgG excretion (mg/24 h)	203 [120–363]	261 [156–404]	184 [97–312]	**0.049**
MAP (mm Hg)	93 [84–103]	96 [85–105]	90 [83–101]	0.125
ACEi/ARB use (%)	60/70 (86)	23/32 (72)	37/38 (97)	**0.010**

α1m, alpha-1-microglobulin; ACEi/ARB, angiotensin-converting-enzyme inhibitor/angiotensin receptor blockers; MAP, mean arterial blood pressure.

Values are given as mean ± SD, median [IQR], number (percentage).

### Anti-PLA2R1ab Level and Prognosis in the Conservative Treatment Arm of the GEMRITUX Trial

In our cohort, baseline aPLA2R1ab titer was not independently associated with disease progression, questioning the value of aPLA2Rab titers as a prognostic biomarker. To confirm our findings, we analyzed the clinical outcome of 26 patients with PLA2R1-associated MN, randomly included in the control arm (with only conservative treatment) of the GEMRITUX clinical trial.^22^ The baseline characteristics of these patients are given in Table 5. Remarkably, aPLA2R1ab titers in this cohort were higher than aPLA2R1ab titers in our study cohort. During 24 months of follow-up, 14 patients were identified as progressors and/or received additional immunosuppressive therapy, whereas 12 patients were identified as nonprogressors, achieving spontaneous remission. Although the median aPLA2R1ab titer at the initiation of the clinical trial was higher in progressors, the difference did not reach significance. The aPLA2R1ab titers in progressors versus nonprogressors are illustrated in Supplementary Appendix Part B Figure S2. In Cox regression analysis, aPLA2R1ab titers below and above the median were not independently associated with disease progression (data not shown).

**Table 5:** Baseline characteristics of patients in the conservative treatment group of the GEMRITUX trial

	Progressors *n* = 14	Nonprogressors *n* = 12	*P* value
Age (y)	61 [41–69]	59 [45–63]	1.000
Sex (M/F, % M)	9/5 (64)	8/4 (67)	1.000
aPLA2R1ab titer (RU/ml)	405 [38–853]	132 [23–465]	0.238
Proteinuria (g/10 mmol)	7.9 [6.4–10.4]	7.1 [3.9–8.9]	0.695
Serum Albumin (g/l)	24 [20–26]	21 [16–23]	0.238
Serum Creatinine (μmol/l)	94 [76–137]	92 [79–109]	0.713

Values are given as median [IQR].

## Discussion

Optimal, personalized management and treatment of patients with PMN require accurate risk prediction. We previously showed that urinary excretion of low molecular weight proteins could be used as a prognostic biomarker, with accuracy comparable to models that included the magnitude and duration of proteinuria.^5^^,^^6^ Still, these models lacked sufficient accuracy, with an AUC of approximately 0.80. The discovery of aPLA2R1ab, which are present in more than 70% of patients with PMN and are likely involved in pathogenesis, offers a new hope for better risk prediction. Indeed, high titers of aPLA2R1ab have been associated with reduced likelihood of spontaneous remission^7^, ^8^, ^9^, ^10^ and poor clinical outcome.^11^^,^^23^^,^^24^ Two studies further suggested that analysis of epitope-specific aPLA2R1ab could improve risk prediction,^13^^,^^14^ although the added value over aPLA2R1ab titer was debated in a recent study.^15^

Literature data on biomarkers for patients with PMN must be interpreted with caution. Most studies were of limited size, and included patients treated and untreated with immunosuppressants. The Food and Drug Administration defines biomarkers and use the BEST (Biomarkers, EndpointS, and other Tools) glossary to define biomarker categories.^16^ With respect to risk prediction, it is important to differentiate between prognostic and predictive biomarkers. A prognostic biomarker is used to identify the likelihood of a clinical event in patients with the disease. Such a biomarker thus predicts the natural course of the disease. In contrast, a predictive biomarker is used to identify individuals who are more or less likely to respond to drug therapy. Distinguishing between prognostic and predictive biomarkers can be difficult. Prognostic biomarkers can be identified from observational data in a natural disease cohort of untreated patients. To identify a predictive biomarker, there should be a comparison between the outcome in treated patients versus controls, as evaluated in clinical trials, both in patients with and without the biomarker. Of note, it is impossible to distinguish between prognostic and predictive biomarkers when only treated patients are included in a study.^16^

Obviously, the study of prognostic biomarkers is relevant only for patients with equivocal outcome. The inclusion of patients who are at no risk for a particular outcome weakens the study. Patients with non-nephrotic PMN have good clinical outcome^25^ and should be excluded from studies that search for prognostic biomarkers. We studied the role of aPLA2R1ab titers as prognostic biomarkers in patients with PMN. The initial analysis included patients with PMN and new-onset or relapsing nephrotic syndrome. However, we observed lower aPLA2R1ab levels in patients with relapsing disease. Therefore, a second analysis including only patients with incident disease was done. Results of the analysis were similar. Immunosuppressive therapy during follow-up was advised for patients with evidence of deteriorating kidney function or patients with severe and/or complicated nephrotic syndrome, who are considered at high risk for progression. We noted clear associations between aPLA2R1ab titers, epitope spreading, and disease severity as reflected by the magnitude of proteinuria, serum albumin levels, and urine excretion of IgG. A Cox proportional hazard model that included UPCR and serum creatinine had reasonable predictive value. This was expected, as proteinuria and estimated glomerular filtration rate are components of the well-known Toronto risk score.^6^ In univariate analysis, higher titers of aPLA2R1ab were associated with disease progression. However, in the proportional hazard model including UPCR and serum creatinine, aPLA2R1ab titers did not improve risk prediction. To confirm our findings, we evaluated the prognostic value of aPLA2R1ab on the risk of disease worsening of patients included in the control arm of the GEMRITUX clinical trial. The GEMRITUX data were similar to those observed in our cohort, with baseline aPLA2R1ab titers having no accurate prognostic value.

Our findings showing that aPLA2R1ab titer has no added value over traditional biomarkers to predict progression seem contradictory to recent studies that also evaluated aPLA2R1ab as a prognostic biomarker. In a previous study, we showed that patients in the highest tertile of aPLA2R1ab were less likely to develop spontaneous remission.^7^ However, accuracy was not reported and multivariable analysis was not done. Other recent studies concluded that patients with low baseline aPLA2R1ab titers were more likely to develop spontaneous remission.^9^^,^^10^ However, these studies have methodological flaws, because they included patients without detectable aPLA2R1ab, and patients without nephrotic syndrome or patients with established renal insufficiency.

In other studies that evaluated the association between aPLA2R1ab and outcome, there was no distinction between aPLA2R1ab as a prognostic and predictive biomarker. All recent studies included patients treated with and without immunosuppressive therapy, and the studies did not report clinical outcomes in the 4 subgroups (treated vs. untreated with and without biomarker). In the largest studies, 85% to 100% of patients were treated.^11^^,^^26^ The study of Mahmud *et al.*,^26^ is an example of a predictive biomarker study. The endpoint in this study, doubling of serum creatinine, or development of end-stage renal disease, was evaluated in treated and untreated patients. In fact, in this study, 85% of nephrotic patients were treated with immunosuppressive therapy; treatment was started on average within 3 months after disease onset, treated patients had higher aPLA2R1ab titers, and were more likely to reach a study endpoint. Thus, this study can be used only to evaluate aPLA2R1ab levels as predictive biomarker (e.g., predicting the efficacy of treatment). Our and these latter studies suggest that aPLA2R1ab titers are more likely to have predictive than prognostic value.

In this study, we also evaluated the added value of “epitope spreading” and our data suggest that epitope spreading might have limited added value over aPLA2R1ab titers.

For clinical practice, it is important to realize that all biomarkers lack accuracy. In discussing treatment options with patients, false negative or false positive rates of 20% to 25% are relevant. Supplementary Appendix Part B Table S4 provides important information for clinical practice. Nephrologists can use this table to discuss individualized prognosis with their individual patient, based on the results of the available assays.

Our study has important limitations. We only evaluated the role of aPLA2R1ab titers at baseline as a prognostic biomarker. It is possible that measurement of changes in aPLA2R1ab has prognostic value. Moreover, measurement of aPLA2R1ab titers may allow to predict response to immunosuppressive therapy.

Another limitation is the inclusion of patients with slightly reduced kidney function. Admittedly, this might have favored serum creatinine as a prognostic biomarker. In a subanalysis that included only patients with a normal kidney function, a wide overlap of aPLA2R1ab levels between progressors and nonprogressors was observed. Also, in a multivariate model including UPCR and serum creatinine, aPLA2R1ab titers did not improve risk prediction.

Another limitation is the definition of disease progression. Doubling of serum creatinine and/or end-stage kidney disease are valid renal endpoints. However, we feel that in the current era it can no longer be accepted to delay immunosuppressive therapy in patients with PMN at high risk for disease progression or complications. Obviously, because the start of immunosuppressive therapy is mostly governed by severity of nephrotic syndrome or kidney dysfunction, its use as study endpoint is debatable and open to confounding. Therefore, the question is if start of immunosuppressive therapy equals progressive disease. We suggest that in our study start of immunosuppressive therapy correctly identified the high-risk patients. We can provide some arguments: First, start of immunosuppressive therapy was not at random but dictated by laboratory markers of kidney disfunction. In 62% of patients, treatment was started after serum creatinine had increased >30% or when estimated glomerular filtration rate was <60 ml/min per 1.73 m^2^. Second, our treatment was restrictive; 35% of patients did not receive immunosuppressive therapy. This is in agreement with reported spontaneous remission rates of 30% to 40 %, suggesting that our clinical decision making was quite reasonable. Third, our treatment was often delayed, with treatment started >6 months after biopsy in 74% of patients. Last, the decision to start therapy was done without knowledge of aPLA2R1ab levels.

One might also debate the endpoint of “nonprogressor.” In this respect, a lack of follow-up in patients who developed a spontaneous remission could pose a problem. If these patients all had a relapse, and would have received immunosuppressive therapy at the time of relapse, there would be no value of predicting prognosis. Therefore, we have gathered follow-up data of patients with spontaneous remission. There were 51 patients who developed spontaneous remission, with a median duration of follow-up after remission of 3.5 [1–8] years. During this period of follow-up, only 2 patients (4%) received immunosuppressive therapy.

We have confirmed our results with data from the control arm of the GEMRITUX clinical trial. Similar to data obtained from the original cohort, aPLA2R1ab titers had no prognostic value in the GEMRITUX patients. A limitation of this validation is obviously the small number of patients. However, these data are of clinical relevance in the individual patient.

Improved outcome prediction will require a more complex model that would include repeated measurements of serum creatinine, UPCR, and aPLA2R1ab titer. Also gender and age should be incorporated into the model. Larger discovery and validation cohorts are needed to develop such risk models/calculators.

## Conclusion

Anti-PLA2R1 antibodies did not improve the risk prediction in PLA2R1-associated PMN when added as a single parameter in a model based on UPCR and serum creatinine.

## Disclosure

GL has patents on the subject of membranous nephropathy (“Diagnostics for membranous nephropathy,” “Methods and kits for monitoring membranous nephropathy,” and “Prognosis and monitoring of membranous nephropathy based on the analysis of PLA2R1 epitope profile and spreading”) with royalties income through CNRS. All the other authors declared no competing interests.

## Supplementary Material

Supplementary File (PDF)

**Supplementary Appendix Part A *N* = 168**

**Table S1.** Risk characteristics of patients according outcome during the 60-month observation period.

**Table S2.** Cox regression analysis: models with known biomarkers for the prediction of progression.

**Table S3.** Cox regression analysis: models adding epitope spreading for the prediction of progression.

**Table S4.** AUC values for all evaluated time horizons for the outcome progression.

**Supplementary Appendix Part B *N* = 156**

**Table S1.** Baseline characteristics of patients according to spreading.

**Table S2.** AUC values for all evaluated time horizons for the outcome progression.

**Table S3.** Cox regression analysis: models adding epitope spreading for the prediction of progression.

**Table S4a+b.** Test characteristics to predict progression in primary MN patients: information based on total duration of follow-up.

**Figure S1.** Calibration plots for the different models.

**Figure S2.** aPLA2R1ab levels at baseline in the GEMRITUX trial of 14 patients with progression and 12 who attained spontaneous remission.

**STROBE Statement.**

## References

[1] McQuarrie E.P., Mackinnon B., Stewart G.A., Geddes C.C. (2010). Membranous nephropathy remains the commonest primary cause of nephrotic syndrome in a northern European Caucasian population. Nephrol Dial Transplant.

[2] Beck L.H., Bonegio R.G., Lambeau G. (2009). M-type phospholipase A2 receptor as target antigen in idiopathic membranous nephropathy. N Engl J Med.

[3] Du Y., Li J., He F. (2014). The diagnosis accuracy of PLA2R-AB in the diagnosis of idiopathic membranous nephropathy: a meta-analysis. PLoS One.

[4] Schieppati A., Mosconi L., Perna A. (1993). Prognosis of untreated patients with idiopathic membranous nephropathy. N Engl J Med.

[5] van den Brand J.A., Hofstra J.M., Wetzels J.F. (2011). Low-molecular-weight proteins as prognostic markers in idiopathic membranous nephropathy. Clin J Am Soc Nephrol.

[6] Cattran D.C., Pei Y., Greenwood C.M. (1997). Validation of a predictive model of idiopathic membranous nephropathy: its clinical and research implications. Kidney Int.

[7] Hofstra J.M., Beck L.H., Beck D.M. (2011). Anti-phospholipase A(2) receptor antibodies correlate with clinical status in idiopathic membranous nephropathy. Clin J Am Soc Nephrol.

[8] Hofstra J.M., Debiec H., Short C.D. (2012). Antiphospholipase A2 receptor antibody titer and subclass in idiopathic membranous nephropathy. J Am Soc Nephrol.

[9] Jullien P., Seitz-Polski B., Maillard N. (2017). Anti-phospholipase A2 receptor antibody levels at diagnosis predicts spontaneous remission of idiopathic membranous nephropathy. Clin Kidney J.

[10] Rodas L.M., Matas-García A., Barros X. (2019). Antiphospholipase 2 receptor antibody levels to predict complete spontaneous remission in primary membranous nephropathy. Clin Kidney J.

[11] Ruggenenti P., Debiec H., Ruggiero B. (2015). Anti-phospholipase A2 receptor antibody titer predicts post-rituximab outcome of membranous nephropathy. J Am Soc Nephrol.

[12] Hoxha E., Thiele I., Zahner G. (2014). Phospholipase A2 receptor autoantibodies and clinical outcome in patients with primary membranous nephropathy. J Am Soc Nephrol.

[13] Seitz-Polski B., Dolla G., Payré C. (2016). Epitope spreading of autoantibody response to PLA2R associates with poor prognosis in membranous nephropathy. J Am Soc Nephrol.

[14] Seitz-Polski B., Debiec H., Rousseau A. (2018). Phospholipase A2 receptor 1 epitope spreading at baseline predicts reduced likelihood of remission of membranous nephropathy. J Am Soc Nephrol.

[15] Reinhard L., Zahner G., Menzel S. (2020). Clinical relevance of domain-specific phospholipase A2 receptor 1 antibody levels in patients with membranous nephropathy. J Am Soc Nephrol.

[16] Food and Drug Administration Context of use. https://www.fda.gov/drugs/cder-biomarker-qualification-program/context-use.

[17] Hofstra J.M., Wetzels J.F. (2012). Management of patients with membranous nephropathy. Nephrol Dial Transplant.

[18] van den Brand J.A., van Dijk P.R., Hofstra J.M., Wetzels J.F. (2014). Long-term outcomes in idiopathic membranous nephropathy using a restrictive treatment strategy. J Am Soc Nephrol.

[19] Floege J., Barbour S., Cattran D. (2019). Management and treatment of glomerular diseases (part 1): conclusions from a Kidney Disease: Improving Global Outcomes (KDIGO) Controversies Conference. Kidney Int.

[20] Manders P., Lutomski J., Smit C. (2018). Radboud Biobank: a central facility for prospective clinical biobanking in the Radboud university medical center, Nijmegen. Open Journal of Bioresources.

[21] Dahnrich C., Komorowski L., Probst C. (2013). Development of a standardized ELISA for the determination of autoantibodies against human M-type phospholipase A2 receptor in primary membranous nephropathy. Clin Chim Acta.

[22] Dahan K., Debiec H., Plaisier E. (2017). Rituximab for severe membranous nephropathy: a 6-month trial with extended follow-up. J Am Soc Nephrol.

[23] Hoxha E., Harendza S., Pinnschmidt H. (2014). M-type phospholipase A2 receptor autoantibodies and renal function in patients with primary membranous nephropathy. Clin J Am Soc Nephrol.

[24] Radice A., Trezzi B., Maggiore U. (2016). Clinical usefulness of autoantibodies to M-type phospholipase A2 receptor (PLA2R) for monitoring disease activity in idiopathic membranous nephropathy (IMN). Autoimmun Rev.

[25] Hladunewich M.A., Troyanov S., Calafati J., Cattran D.C. (2009). The natural history of the non-nephrotic membranous nephropathy patient. Clin J Am Soc Nephrol.

[26] Mahmud M., Pinnschmidt H., Reinhard L. (2019). Role of phospholipase A2 receptor 1 antibody level at diagnosis for long-term renal outcome in membranous nephropathy. PLoS One.

