# Supplementary material for: Anti-PLA2R1 Antibodies as Prognostic Biomarker in Membranous Nephropathy
Source: Kidney Int Rep. 2021 Apr 22;6(6):1677–86. doi: 10.1016/j.ekir.2021.04.002 (PMC8207302; doi:10.1016/j.ekir.2021.04.002)
Supplement: Supplementary File (PDF) [file mmc1.pdf]

Supplementary Tables

Supplementary Appendix part A: N=168 full cohort

Table S1: Risk characteristics of patients according to outcome during the 60 months observation period

**Clinical characteristics**	**Overall (n=168)**	**Progressors N=110**	**Remission N=54**	**Persistent NS N=4****	**p-value**
Age (years)	52 ± 13	53 ± 13	51 ± 14	45 [40-61]	0.525
Gender (M/F, % M)	118/50 (70)	74/36 (67)	40/14 (74)	4/0 (100)	0.281
Previous disease episode (n(%))	10 (6)	8 (7)	2 (4)	0 (0)	0.582
Previous IS treatment (n(%))	7 (4)	5 (5)	2 (4)	0 (0)	0.886
Time from biopsy (months)	2.5 [1.2-7.8]	2.4 [1.2-6.8]	3.0 [1.4-8.1]	9.9 [0.9-31]	0.418
Serum creatinine (µmol/L)	92 ± 18	95 ± 20	86 ± 15	94 [69-116]	**0.020**
Serum albumin (g/L)	21 ± 5	20 ± 5	23 ± 5	24 [21-28]	**0.001**
Serum cholesterol (mmol/L)	6.5 [5.2-8.5]	7.2 [5.4-9.2]	5.8 [5.2-7.1]	6.0 [5.0-13.0]	**0.006**
Serum IgG (g/L)	4.4 [3.2-5.5]	4.0 [2.9-5.1]	5.1 [4.2-6.2]	4.0 [4.0-8.0]	**0.002**
Proteinuria (g/10 mmol)	7.2 [5.6-10.7]	8.8 [6.2-11.3]	5.9 [4.5-7.8]	5.5 [4.0-7.0]	**0.000**
aPLA2R1ab titer Euroimmun (RU/mL)	106 [45-230]	127 [66-306]	49 [31-127]	217 [39-829]	**0.000**
Spreaders (n(%))	116 (69)	85 (77)	29 (54)	2 (50)	**0.006**
Urinary β2m (ng/min)	1016 [282-3264]	2258 [481-7031]	315 [164-794]	348 [126-8743]	**0.000**
Urinary α1m (µg/min)	45 [27-81]	62 [39-101]	30 [20-43]	13 [8-57]	**0.000**
IgG excretion (mg/24 hours)	306 [158-520]	398 [231-567]	186 [108-315]	90 [60-309]	**0.000**
MAP (mmHg)	93 [86-102]	93 [87-103]	90 [83-101]	95 [91-105]	0.161
ACEi/ARB use (%)	114/133 (85)	66/84 (79)	44/45 (98)	4/4 (100)	**0.004**

Values are given as Mean ± SD, Median [IQR], number (percentage). *Time from biopsy= interval between biopsy and standardized measurement in our hospital. **In patients with persisting proteinuria (N=4) values are given as Median [Range].

NS= nephrotic syndrome, MAP= mean arterial blood pressure, ACEi/ARB= angiotensin-converting-enzyme inhibitor/angiotensin receptor blockers.

Table S2: Cox-regression analysis: models with known biomarkers for the prediction of progression

	AUC 36 months	HR	95 % CI
**Model 1**			
Screat	0.697	1.019	1.007-1.030
UPCR		1.176	1.110-1.246
**Model 2**			
Screat	0.689	1.018	1.007-1.029
UPCR		1.168	1.100-1.239
Log.aPLA2R1ab		1.256	0.844-1.867

AUC= area under the curve/C-statistic, HR= hazard ratio, CI= confidence interval, Screat= serum creatinine, UPCR= protein-creatinine ratio in 24-hours urine, Log.aPLA2R1ab= natural log of anti-PLA2R antibodies.

Table S3: Cox-regression analysis: models adding epitope spreading for the prediction of progression

	AUC 36 months	HR	95 % CI
**Model 1**			
Screat	0.697	1.019	1.007-1.030
UPCR		1.176	1.110-1.246
**Model 2**			
Screat	0.701	1.018	1.007-1.029
UPCR		1.167	1.101-1.237
Epitope spreading		1.592	1.014-2.499

AUC= area under the curve/C-statistic, HR= hazard ratio, CI= confidence interval, Screat= serum creatinine, UPCR= protein-creatinine ratio in 24-hours urine.

Table S4: AUC values for all evaluated time horizons for the outcome progression

Model 1: Screat and UPCR

Time	AUC	95 % CI
6	0.735	0.655-0.815
12	0.694	0.610-0.778
18	0.691	0.602-0.780
24	0.702	0.605-0.800
36	0.697	0.564-0.829
60	0.727	0.507-0.946

Model 2: Screat, UPCR and Log.aPLA2R1ab

Time	AUC	95 % CI
6	0.737	0.658-0.816
12	0.693	0.610-0.778
18	0.690	0.601-0.779
24	0.692	0.592-0.792
36	0.689	0.557-0.822
60	0.709	0.479-0.940

Model 3: Screat, UPCR and epitope spreading

Time	AUC	95 % CI
6	0.757	0.681-0.833
12	0.705	0.621-0.788
18	0.710	0.621-0.799
24	0.720	0.617-0.823
36	0.701	0.553-0.849
60	0.745	0.499-0.991

Model 4: Screat, UPCR, Log.aPLA2R1ab and epitope spreading

Time	AUC	95 % CI
6	0.755	0.679-0.832
12	0.705	0.622-0.789
18	0.709	0.620-0.800
24	0.718	0.614-0.821
36	0.701	0.555-0.848
60	0.745	0.496-0.994

Time= time between the first standardized measurement and the start of immunosuppressive treatment , AUC= area under the curve/C-statistic, CI= confidence interval, Screat= serum creatinine, UPCR= protein-creatinine ratio in 24-hours urine, Log.aPLA2R1ab= natural log of anti-PLA2R1 antibodies.

Supplementary Appendix part B: N=156 incident patients

Table S1: baseline characteristics of patients according to spreading

**N=156**	**Non-spreaders** N=44	**Spreaders** N=112	**p-value**
Age	51 ± 11	52 ± 13	0.688
Gender (M/F, % M)	36/8 (82)	76/36 (68)	0.113
Disease duration (months)	3.0 [1.6-8.1]	2.1 [1.1-6.2]	0.374
Serum creatinine (µmol/L)	87 [82-95]	94 [81-109]	0.056
Serum albumin (g/L)	23 [19-27]	19 [16-24]	**0.008**
Serum cholesterol (mmol/L)	6.5 [4.7-8.3]	6.8 [5.4-9.2]	0.374
aPLA2R1ab titer Euroimmun (RU/mL)	54 [30-105]	147 [56-297]	**0.000**
Serum IgG (g/L)	4.7 [3.8-5.5]	4.2 [3.0-5.4]	0.594
Proteinuria (g/10 mmol)	6.3 [4.9-10.7]	7.4 [5.9-11.0]	0.109
Urinary β2M (ng/min)	418 [189-2213]	1469 [316-5344]	**0.031**
Urinary α1M (ug/min)	35 [20-72]	52 [30-89]	0.120
IgG excretion (mg/24 hours)	245 [112-442]	311 [185-535]	0.372
MAP (mmHg)	95 [88-104]	91 [84-102]	0.253
ACEi/ARB use (n(%))	31/37 (84)	74/87 (85)	0.061

Values are given as Mean ± SD, Median [IQR]

MAP= mean arterial blood pressure, ACEi/ARB= angiotensin-converting-enzyme inhibitor/angiotensin receptor blockers.

Table S2: AUC values for all evaluated time horizons for the outcome progression

Model 1: Screat and UPCR

Time	AUC	95 % CI
6	0.760	0.681-0.839
12	0.719	0.635-0.804
18	0.714	0.625-0.804
24	0.728	0.632-0.825
36	0.715	0.587-0.842
60	0.752	0.542-0.962

Model 2: Screat, UPCR and Log.aPLA2R1ab

Time	AUC	95 % CI
6	0.760	0.682-0.839
12	0.720	0.636-0.805
18	0.718	0.629-0.808
24	0.716	0.616-0.816
36	0.708	0.581-0.834
60	0.719	0.490-0.948

Model 3: Screat, UPCR and epitope spreading

Time	AUC	95 % CI
6	0.783	0.709-0.857
12	0.735	0.652-0.817
18	0.734	0.644-0.824
24	0.742	0.639-0.846
36	0.723	0.580-0.867
60	0.765	0.523-1.000

Model 4: Screat, UPCR, Log.aPLA2R1ab and epitope spreading

Time	AUC	95 % CI
6	0.781	0.707-0.856
12	0.734	0.651-0.817
18	0.732	0.641-0.822
24	0.737	0.632-0.841
36	0.718	0.576-0.860
60	0.756	0.516-0.996

Time= time between the first standardized measurement and the start of immunosuppressive treatment , AUC= area under the curve/C-statistic, CI= confidence interval, Screat= serum creatinine, UPCR= protein-creatinine ratio in 24-hours urine, Log.aPLA2R1ab= natural log of anti-PLA2R1 antibodies.

Table S3: Cox-regression analysis: models adding epitope spreading for the prediction of progression

	AUC 36 months	HR	95 % CI
**Model 1**	0.715		
Screat		1.019	1.007-1.031
UPCR		1.185	1.116-1.258
**Model 2**	0.723		
Screat		1.018	1.007-1.030
UPCR		1.178	1.109-1.251
Epitope spreading		1.727	1.055-2.828

AUC= area under the curve/C-statistic, HR= hazard ratio, CI= confidence interval, Screat= serum creatinine, UPCR= protein-creatinine ratio in 24-hours urine.

Table S4: **Test characteristics to predict progression in primary MN patients: information based on total duration of follow-up.**

Table 4a: N=156

Threshold value	Sensitivity	Specificity	Accuracy	N
**aPLA2R1ab titer** (RU/mL)				
>50	82	50	71	111
>100	64	63	64	86
>150	51	81	61	63
>200	42	88	58	50
>250	33	94	53	37
>300	29	94	51	33
>350	20	94	45	24
>400	16	96	43	19

Explanation of the Table:

This Table provides information that can be used to discuss treatment options with the patients based on a prediction of the prognosis guided by the patient's risk profile.

How to use this Table? Some examples may help. Assume a patient with an aPLA2R1ab level of 60 RU/ml. The 2 by 2 table based on our cohort of 156 patients will be:

	No progression	Progression	**Total**
aPLA2R1ab (RU/ml) < 50	26 (TN)	19 (FN)	45
aPLA2R1ab (RU/ml) > 50	26 (FP)	85 (TP)	111
**Total**	52	104	156

TN= true negative, FN= false negative, FP= false positive, TP= true positive.

Thus the predicted risk of progression is 85/111=77%; the risk for this patient to receive unnecessary treatment is 23%. If this patient had an aPLA2R1ab level < 60 RU/ml, the risk of progression would be lower (19/45=42%). The risk of unnecessary treatment amounts 58 %.

If the patient has an aPLA2R1-ab level of 210 RU/ml, the risk of progression would be higher at 88% (44/50), thus the risk of unnecessary treatment decreases.

	No progression	Progression	**Total**
aPLA2R1ab (RU/ml) < 200	46 (TN)	60 (FN)	106
aPLA2R1ab (RU/ml) > 200	6 (FP)	44 (TP)	50
**Total**	52	104	156

TN= true negative, FN= false negative, FP= false positive, TP= true positive.

When calculating positive predictive value (PPV) it is important to realize that this value is dependent on the baseline risk of a population. A biomarker with the same calculated sensitivity and specificity will be less valuable when applied to a population with much lower baseline risk.

Of note, the use of a fixed cutoff value has disadvantages in individualized patient care. An example is given using different quintiles of aPLA2R1ab levels showing that the positive predictive value (PPV) increases at higher levels.

	Positive predictive value
aPLA2R1ab level <40 RU/ml	48 %
aPLA2R1ab level 40-77 RU/ml	52 %
aPLA2R1ab level 78-152 RU/ml	66 %
aPLA2R1ab level 152-307 RU/ml	77 %
aPLA2R1ab level >307 RU/ml	87 %

Table 4b: N=85 patients with normal serum creatinine. See above for explanations.

**Threshold value**	**Sensitivity**	**Specificity**	**Accuracy**	**N**
**aPLA2R1ab titer** (RU/mL)				
>50	85	52	67	55
>100	59	61	60	41
>150	44	80	64	26
>200	36	89	65	19
>250	26	93	62	13
>300	26	93	62	13
>350	18	93	59	10
>400	15	96	59	8

	Positive predictive value
aPLA2R1ab level <39 RU/ml	24 %
aPLA2R1ab level 39-68 RU/ml	29%
aPLA2R1ab level 69-115 RU/ml	59 %
aPLA2R1ab level 125-209 RU/ml	47 %
aPLA2R1ab level >210 RU/ml	71 %

Figure S1: Calibration plots for the different models

Model 1:

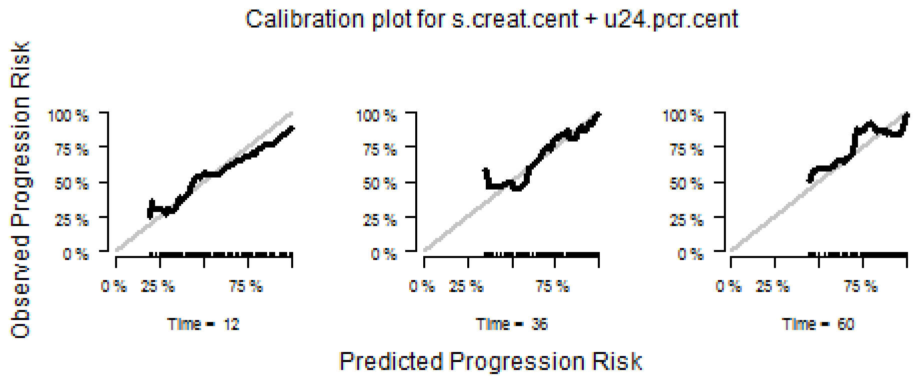

Model 2:

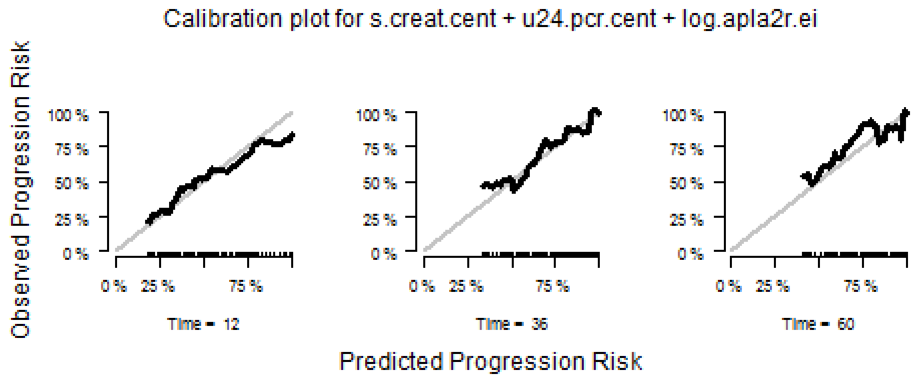

Model 3:

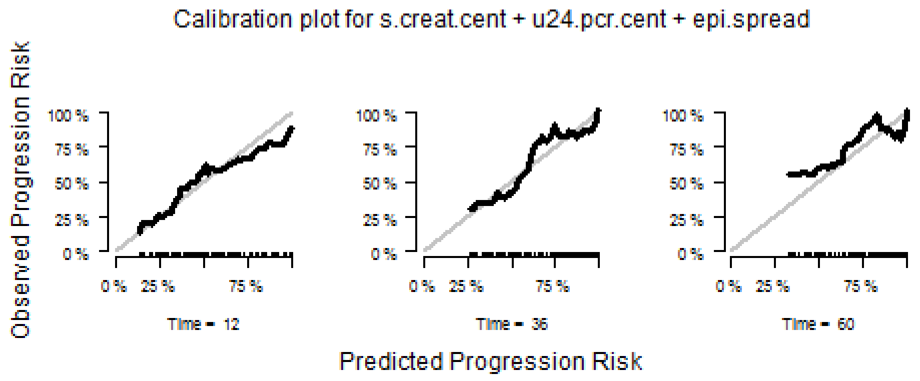

Figure S2: aPLA2R1ab levels at baseline in the GEMRITUX trial of 14 patients with progression and 12 who attained spontaneous remission

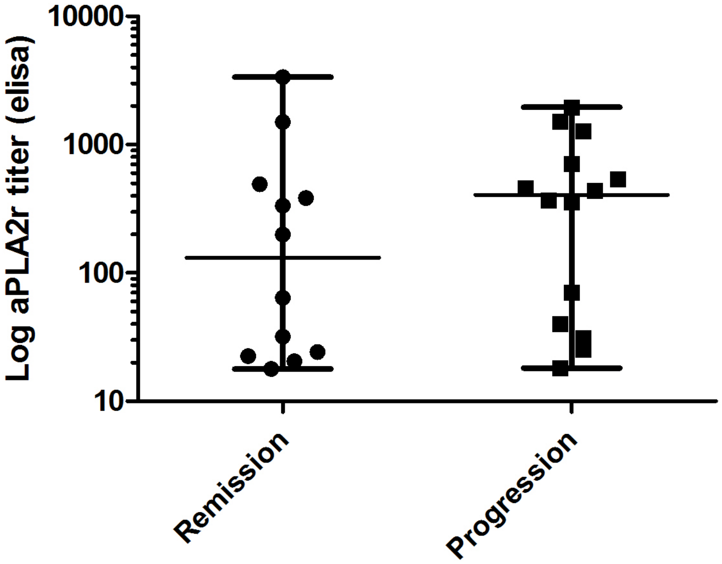

Modified STROBE Statement—checklist of items that should be included in reports of observational studies (Cohort/Cross-sectional and case-control studies)

We have addresses all these issues in our manuscript

	Item No	Recommendation
**Title and abstract**	1	(*a*) Indicate the study's design with a commonly used term in the title or the abstract
		(*b*) Provide in the abstract an informative and balanced summary of what was done and what was found
**Introduction**		
Background/rationale	2	Explain the scientific background and rationale for the investigation being reported
Objectives	3	State specific objectives, including any prespecified hypotheses
**Methods**		
Study design	4	Present key elements of study design early in the paper
Setting	5	Describe the setting, locations, and relevant dates, including periods of recruitment, exposure, follow-up, and data collection
Participants	6	(*a*) *Cohort study*—Give the eligibility criteria, and the sources and methods of selection of participants. Describe methods of follow-up<br>*Case-control study*—Give the eligibility criteria, and the sources and methods of case ascertainment and control selection. Give the rationale for the choice of cases and controls<br>*Cross-sectional study*—Give the eligibility criteria, and the sources and methods of selection of participants
Variables	7	Clearly define all outcomes, exposures, predictors, potential confounders, and effect modifiers. Give diagnostic criteria, if applicable
Data sources/ measurement	8*	For each variable of interest, give sources of data and details of methods of assessment (measurement).
Bias	9	Describe any efforts to address potential sources of bias
Study size	10	Explain how the study size was arrived at (if applicable)
Quantitative variables	11	Explain how quantitative variables were handled in the analyses. If applicable, describe which groupings were chosen and why

Statistical methods	12	(*a*) Describe all statistical methods, including those used to control for confounding
		(*b*) Describe any methods used to examine subgroups and interactions
		(*c*) Explain how missing data were addressed
		(*d*) *Cohort study*—If applicable, explain how loss to follow-up was addressed<br>*Case-control study*—If applicable, explain how matching of cases and controls was addressed<br>*Cross-sectional study*—If applicable, describe analytical methods taking account of sampling strategy
		(*e*) Describe any sensitivity analyses
**Results**		
Participants	13*	(a) Report numbers of individuals at each stage of study—eg numbers potentially eligible, examined for eligibility, confirmed eligible, included in the study, completing follow-up, and analyzed
		(c) **Use of a flow diagram**
Descriptive data	14*	(a) Give characteristics of study participants (eg demographic, clinical, social) and information on exposures and potential confounders
		(b) Indicate number of participants with missing data for each variable of interest
		(c) *Cohort study*—Summarise follow-up time (eg, average and total amount)
Outcome data	15*	*Cohort study*—Report numbers of outcome events or summary measures over time
		Case-control study—Report numbers in each exposure category, or summary measures of exposure
		Cross-sectional study—Report numbers of outcome events or summary measures
Main results	16	(*a*) Give unadjusted estimates and, if applicable, confounder-adjusted estimates and their precision (eg, 95% confidence interval). Make clear which confounders were adjusted for and why they were included
Other analyses	17	Report other analyses done—eg analyses of subgroups and interactions, and sensitivity analyses

**Discussion**		
Key results	18	Summarise key results with reference to study objectives
Limitations	19	Discuss limitations of the study, taking into account sources of potential bias or imprecision. Discuss both direction and magnitude of any potential bias
Interpretation	20	Give a cautious overall interpretation of results considering objectives, limitations, multiplicity of analyses, results from similar studies, and other relevant evidence
Generalisability	21	Discuss the generalisability (external validity) of the study results

*Give information separately for cases and controls in case-control studies and, if applicable, for exposed and unexposed groups in cohort and cross-sectional studies.

Note: An Explanation and Elaboration article discusses each checklist item and gives methodological background and published examples of transparent reporting. The STROBE checklist is best used in conjunction with this article (freely available on the Web sites of PLoS Medicine at http://www.plosmedicine.org/, Annals of Internal Medicine at http://www.annals.org/, and Epidemiology at http://www.epidem.com/). Information on the STROBE Initiative is available at www.strobe-statement.org.
